# Recall of intestinal helminthiasis by HIV-infected South Africans and avoidance of possible misinterpretation of egg excretion in worm/HIV co-infection analyses

**DOI:** 10.1186/1471-2334-6-88

**Published:** 2006-05-26

**Authors:** Vera J Adams, Miles B Markus, Zilungile L Kwitshana, Muhammad A Dhansay, Lize van der Merwe, Gerhard Walzl, John E Fincham

**Affiliations:** 1 Nutritional Intervention Research Unit, P O Box 19070, Tygerberg 7505, South Africa; 2 University of the Witwatersrand, Johannesburg 2050, South Africa; 3 Nutritional Intervention Research Unit, P O Box 70380, Durban-Overport 4067, South Africa; 4 Biostatistics Unit, Medical Research Council of South Africa, P O Box 19070, Tygerberg 7505, South Africa; 5 Department of Medical Biochemistry, University of Stellenbosch, Tygerberg 7505, South Africa

## Abstract

**Background:**

Ascariasis and HIV/AIDS are often co-endemic under conditions of poverty in South Africa; and discordant immune responses to the respective infections could theoretically be affecting the epidemic of HIV/AIDS in various ways. It is well-known that sensitisation to helminthic antigens can aggravate or ameliorate several non-helminthic diseases and impair immunisation against cholera, tetanus and tuberculosis. The human genotype can influence immune responses to *Ascaris *strongly. With these factors in mind, we have started to document the extent of long-term exposure to *Ascaris *and other helminths in a community where HIV/AIDS is highly prevalent. In more advanced studies, objectives are to analyse relevant immunological variables (e.g. cytokine activity and immunoglobulin levels). We postulate that when *Ascaris *is hyperendemic, analysis of possible consequences of co-infection by HIV cannot be based primarily on excretion vs non-excretion of eggs.

**Methods:**

Recall of worms seen in faeces was documented in relation to the age of adult volunteers who were either seropositive (n = 170) or seronegative (n = 65) for HIV. Reasons for HIV testing, deworming treatments used or not used, date and place of birth, and duration of residence in Cape Town, were recorded. Confidence intervals were calculated both for group percentages and the inter-group differences, and were used to make statistical comparisons.

**Results:**

In both groups, more than 70% of participants were aware of having passed worms, often both when a child and as an adult. Most of the descriptions fitted *Ascaris*. Evidence for significantly prolonged exposure to helminthic infection in HIV-positives was supported by more recall of deworming treatment in this group (p < 0.05). Over 90% of the participants had moved to the city from rural areas.

**Conclusion:**

There was a long-term history of ascariasis (and probably other helminthic infections) in both of the groups that were studied. In women in the same community, and in children living where housing and sanitation are better, *Ascaris *sero-prevalence exceeded egg-prevalence by two- and three-fold, respectively. For ongoing and future analyses of possible consequences of co-infection by *Ascaris *(and/or other helminths) and HIV/AIDS (and/or other bystander conditions), comparisons must be based mainly on disease-related immunological variables. Especially in adults, comparisons cannot be based only on the presence or absence of eggs in excreta.

## Background

Some literature reviews and research studies have suggested that immunological responses to frequent challenge from helminth parasites and other pathogens may be influencing rates of infection by HIV and/or progression to AIDS in developing countries [1,2]. Furthermore, it is likely that the efficacy of anti-HIV vaccines will be impaired under these conditions [3-5]. Helminthiasis is usually characterised by a strong type 2 immune profile [6-8], which can down-regulate an anti-viral type 1 reaction; and a study has shown that a balanced type 1/type 2 response was significantly related to long-term non-progression of HIV infection [9]. When the maternal immune profile has been imbalanced by helminthic infection, the risk of transmitting HIV to babies appears to increase [10]. Theories about immunological relationships between infection by worms and HIV/AIDS are supported by the reality of this kind of interaction between helminthiasis and immune-mediated disease associated with the human T cell lymphotropic virus type 1 (HTLV-1), which like HIV is a retrovirus [11].

In many South African communities, high population densities, poor living conditions and deficient public health service delivery support ongoing challenge from various infections, with *Ascaris *and HIV as components of the milieu [1,12-19]. The prevailing environmental conditions promote involuntary ingestion of *Ascaris *eggs, which either leads to re-infection (the lifespan of *Ascaris *is relatively short compared to that of other common helminths [20]) or ensures ongoing antigenic challenge. Most human adults are probably in a state of immunological holoendemic equilibrium [21] that reduces patent infection by *Ascaris*. In children, infection by *Ascaris *has been shown to polarise the immune profile towards type 2, which is associated with an impaired response to a cholera vaccine [22,23]. These *Ascaris*-specific effects were reversed by deworming with albendazole [24], which might be an inexpensive way to improve the efficacy of potential vaccines against HIV [1-3] and other diseases [1,4,23-25].

In view of widespread co-endemicity of *Ascaris *and HIV/AIDS in South Africa, our first objective is to begin to define the lifespan history of infection challenge from *Ascaris *in people living in communities where HIV/AIDS is a growing problem. If lifelong challenge is generally present, further research would aim to ascertain which immunological and/or genetic variables are better than the temporary presence or absence of *Ascaris *eggs in faeces, for assessing the potential for disease interaction. We are aware of more than 20 recent studies that have used faecal helminth egg-positivity or egg-negativity as the main or only worm-related variable in comparisons relating to HIV infection (and/or other bystander diseases) in areas where helminths are highly endemic. We contend that this approach is fundamentally flawed for reasons that will be discussed. Understanding would improve if analysis were to be based on variables that include specific immunoglobulins [6,8,26-28], cytokine ratios [6-9,29-33], eosinophilia [6,8,13] and genetic markers [34-37]. If significant disease interaction can be confirmed by the use of more reliable variables, the overall objective is to then make recommendations on how implementation of prevention and control of ascariasis and other helminthic infections might influence the epidemic of HIV/AIDS [1,2], anti-HIV vaccine testing and the efficacy of immunisation (if vaccines can be produced) [1-3,5].

The present study is based mainly on people who have moved from rural districts in the Eastern Cape province (EC) to a suburb of Cape Town (CT). HIV/AIDS is a major epidemic in both of these environments [17-19]. The only way to get the history of infestation by worms in the rural areas from which the people came, was by personal recall of helminths seen in faeces. This is a reasonable approach because *Ascaris *is so large [20], and is especially conspicuous when availability of toilets is limited, as in some densely-populated rural areas and urban slums [12,14,15]. The frequency of recall of probable *Taenia *segments has also been documented but discussion is restricted mainly to *Ascaris*. However, taeniasis and cysticercosis (including neurocysticercosis) are well-known in both areas [38,39]. Since participation in the study was voluntary and no deliberate selection was imposed, it is probable that the participants were representative of most adults, especially women, living under similar circumstances. In the CT community, recent surveys found that 24.9% (95% CI 20.7 – 29.1) of women attending antenatal clinics were infected by HIV [17]; and more than 75% of pupils at 12 primary schools had *Ascaris *eggs in their faeces [12]. Stigma (and fear) associated with a positive HIV test is a major problem that affects the design of research in communities where the prevalence of HIV/AIDS is high [40]. Consequently, pragmatic, non-random, exploratory projects, such as the one now reported, are sometimes the only way to start research.

## Methods

This report covers the first set of results from a project that was approved by the Ethics Committees of the South African Medical Research Council and the University of Stellenbosch, South Africa, under the overall title: *Tracking the immune profile of adults who are either HIV-positive or HIV-negative, during helminthic infection and anthelmintic treatment*.

An HIV/AIDS support group in the CT suburb of Khayelitsha was approached about participation in an initial survey. Information sessions to explain the objectives of the research and to answer questions, were run by AIDS counsellors serving the support group. After comprehensive discussion in the vernacular language (isiXhosa), 170 HIV-positive and 65 HIV-negative adults volunteered to participate and signed informed consent forms. These documents and completed questionnaires are on file with the Helminth Section of the Nutritional Intervention Research Unit, Medical Research Council, CT, South Africa. The consent given included permission to confirm HIV status by re-testing.

Retrospective, enquiry-based research was the only way to obtain lifespan information on the history of infection by worms (starting in childhood) in the adult volunteers, as well as demographic details defining the extent of relocation to CT and the memory of anthelmintic treatment or non-treatment. A semi-structured questionnaire was drafted in English, translated into Xhosa, and back-translated into English to confirm that meanings were conveyed accurately. It was tested by Xhosa-speakers for logic, accuracy and fluency. Adjustments were made as required. Xhosa-speaking AIDS counsellors interviewed individual volunteers and recorded their answers to the final questions. Replies regarding infection by worms were used to ascertain: (a) if the participant was aware of ever having passed worms in any way (particularly in faeces), as a child and/or as an adult; (b) the estimated age when worms were noticed as a child and/or as an adult; and (c) the appearance of worms seen, as well as the kind of conventional or traditional medicine that was used for treatment, if any. Date and place of birth were confirmed from identity documents and used to determine age. Information on length of residence in the Khayelitsha suburb of CT was also obtained.

Wilson's direct method was used to set 95% confidence intervals (CI) for the percentages shown in the tabulated results [41]. Differences between percentages for HIV-positives and HIV-negatives were assessed for significance by calculating the 95% CI for the net value by Newcombe's method [41]. When this CI excludes zero, it indicates a significant difference (p < 0.05). Ages and duration of residence in CT were compared by means of heterosedastic two-sided t-tests, assuming unequal variance.

## Results

### HIV/AIDS status

The original motivations for HIV testing are summarised in Table 1. More than 40% of the HIV-positive group were tested because they had a clinical condition that can be AIDS-associated, whereas more than 75% of the HIV-negative group had volunteered for testing on the basis that they preferred to know their status.

**Table 1:** Original reasons for HIV testing

**HIV-positive (n = 170)**	**Numbers**	**Percentage and 95% CI**
Possible AIDS-associated condition	72/170	42.3 (36.3, 48.7)
Prevention of mother-to-child transmission	62/170	36.5 (29.6, 43.9)
High-risk behaviour, counselled to test	36/170	21.2 (15.7, 27.9)

**HIV-negative (n = 65)**		

Possible AIDS-associated condition	9/65	13.9 (7.5, 24.3)
A condition not usually AIDS-associated	6/65	9.2 (4.3, 18.7)
Prevention of mother-to-child transmission	1/65	1.5 (0.3, 8.2)
Voluntary testing	49/65	75.4 (63.7, 84.2)

CI = confidence interval.

### Demography

The percentage distributions by place of birth, with 95% confidence intervals, are shown in Table 2. The HIV-positive volunteers consisted of 143 women and 27 men (n = 170), i.e. 84.1% women. The HIV-negative volunteers comprised 52 women and 13 men (n = 65), i.e. 80.0% women. More than 91% of the HIV-positives and more than 95% of the HIV-negatives were not born in CT but all the participants were South Africans.

**Table 2:** Place of birth of volunteers

	**HIV-positives (n = 170)**^2^			**HIV-negatives (n = 65)**^2^		
**Place of Birth**	**Numbers**	**%**	**95% CI**	**Numbers**	**%**	**95% CI**

City of Cape Town	15/170	8.8	5.4, 14.0	3/65	4.6	1.6, 12.7
Western Cape province^1^	1/170	0.6	0.1, 3.3	0/65	0	0.0, 5.6
Eastern Cape province	151/170	88.8	83.2, 92.7	61/65	93.9	85.2, 97.6
Northern Cape province	1/170	0.6	0.1, 3.3	0/65	0	0.0, 5.6
Gauteng province	1/170	0.6	0.1, 3.3	1/65	1.5	0.3, 8.2
Mpumalanga province	1/170	0.6	0.1, 3.3	0/65	0	0.0, 5.6

CI = confidence interval.

^1^Excluding the city of Cape Town.

^2^There were no statistically significant differences between the percentages of HIV-positives and HIV-negatives by place of birth.

### Age when questioned, duration of residence in CT and worm infection as children and adults

The adults in the HIV-positive group were younger than those in the HIV-negative group and had lived in CT for a shorter period (p < 0.01, Table 3). For individuals who were not born in CT (> 91%), the data indicate that worm infection as a child occurred mainly before relocation to the city; and as an adult, was after the move. This deduction is supported by the close concordance of the within-group means and medians for the estimated ages at which participants remembered passing worms when children or adults, respectively. There was no significant difference between the groups in mean age at the time of childhood or adult infection. The data suggest that the HIV-positive adults may have seen worms more recently than the HIV-negative adults.

**Table 3:** Age when questioned, duration of residence in Cape Town and worm infection at estimated child and adult ages

**HIV-positive (n = 169)**^1^	**Age when questioned**	**Duration**^2^	**Worms at child age**^3^	**Worms at adult age**^4^
Mean age	30.6*	9.5^†^	9.2 ^NSD^	26.6 ^NSD^
Median age	28.7	8.1	9.0	28.0
Age range	16.4 – 63.1	0.1 – 40.6	4.0 – 14.0	15.0 – 42.0

**HIV-negative (n = 63)**^1^	**Age when questioned**	**Duration**^2^	**Worms at child age**^3^	**Worms at adult age**^4^

Mean age	39.9*	13.5†	10.3 ^NSD^	28.9 ^NSD^
Median age	41.6	12.5	10.5	25.0
Age range	15.2 – 74.5	0.1 – 54.1	5.0 – 14.0	15.0 – 49.0

^1^Numbers reduce from 170 and 65 because birth dates were not known for one HIV-positive and two HIV-negative individuals.

^2^Duration of residence in Cape Town (years).

^3^Child = up to 15^th ^birthday. Infection as a child was remembered by 91 HIV-positives and 26 HIV-negatives.

^4^Adult = after 15^th ^birthday. Infection as an adult was remembered by 25 HIV-positives and 16 HIV-negatives.

*Mean ages when questioned were significantly different (p < 0.01).

^†^Mean durations of stay in Cape Town were significantly different (p < 0.01).

NSD = not significantly different.

### Recall of worm infestation

More than 70% of participants in both of the groups were aware that they had been infected by worms as a child and/or as an adult (Table 4). A significantly greater percentage of HIV-positives remembered having had worms both when they were children and adults (p < 0.05), which may indicate more sequential ascariasis (and probably other helminthic infections) in this group.

**Table 4:** Comparison between percentages of volunteers who remembered being infected by worms

	**HIV-positives**			**HIV-negatives**			**Comparison of %**	
**Recall of worms**^1^	**Numbers**	**%**	**95% CI**	**Numbers**	**%**	**95% CI**	**Difference**	**95% CI**^4^

At any age	122/167^2^	73.1	65.9, 79.2	51/65	78.5	67.0, 86.7	-5.4	-16.4, 7.6
As a child	99/167^2^	59.3	51.7, 66.4	31/65	47.7	36.0, 59.6	11.6	-2.5, 25.3
As a child and as an adult	47/165^3^	28.5	22.1, 38.5	8/65	12.3	6.4, 22.5	16.2*	4.2, 25.6
As an adult (only)	70/165^3^	42.4	35.1, 50.1	28/65	43.1	31.8, 55.2	-0.7	-14.8, 13.0
No recall of worms	45/167^2^	26.9	20.8, 34.1	14/65	21.5	13.3, 33.0	5.4	-7.6, 16.4

CI = confidence interval.

^1^Child = up to 15^th ^birthday. Adult = after 15^th ^birthday.

^2^The reduction from n = 170 is because three individuals were uncertain about whether they had, or had not, seen worms in faeces; hence, n = 167.

^3^Of the 167 who gave definitive answers on infection, two knew that they had been infected as children but were not sure if they had been infected when adults; hence, n = 165.

^4^CIs which exclude zero indicate that the HIV-positives and HIV-negatives were significantly different (p < 0.05) [41].

*Significantly more HIV-positives remembered having been infected by worms as both a child and an adult (p < 0.05).

### Probable identity of worms seen

Relatively reliable identification of helminths in faeces would pertain to *Ascaris *because the worms are big, conspicuous and sometimes numerous [20]. Descriptions that combined "long" or "big" with "white", "whitish" and "pointed" were interpreted as referring to *Ascaris*, which is highly prevalent in CT and the Western Cape province (WC) [12-14], as well as in the EC [15]. *Taenia *tapeworm infection is common in some districts in the EC [38,39], from where most of the participants originated (Table 2). The terms "flat" combined with "small", "white" or "whitish" probably referred mainly to *Taenia *segments passed in faeces. The frequency of descriptions that fitted either *Ascaris *or *Taenia *respectively, were not significantly different between the HIV-positive and HIV-negative groups (Table 5). In the absence of "flat", "small" might refer to *Enterobius *(1 case), or hookworm when the colour was given as red, as after a blood meal (1 case).

**Table 5:** Frequency of credible descriptions of either *Ascaris *worms or *Taenia *segments in faeces

		***Ascaris *worms**			***Taenia *segments**		
Group	Descriptions^1^	n	%^2^	95% CI	n	%^2^	95% CI

HIV-positive	103	86	83.5	(75.1, 89.4)	17	16.5	(10.6, 24.9)
HIV-negative	42	35	83.3	(69.4, 91.7)	6	14.3	(6.2, 27.8)

CI = confidence interval.

^1^Descriptions of worms were given by 103 and 42 individuals in the respective groups.

^2^The differences between the percentages for the two groups are not significant for either *Ascaris *worms or *Taenia *segments.

### Treatment used against worms and non-treatment

Recall of deworming treatment with conventional or traditional medicines, or of non-treatment, is summarised in Table 6. Significantly more HIV-positives than HIV-negatives remembered deworming treatment and fewer positives thought it was not necessary to treat against worms (p < 0.05). These results suggest significantly more worm infestation in the HIV-positives, both as adults and during childhood (Table 4).

**Table 6:** Comparison between the percentages of volunteers who remembered deworming treatment or non-treatment

	**HIV-positives (n = 170)**			**HIV-negatives (n = 65)**			**Comparison**	
**Treatment or non-treatment**	**Numbers**^2^	**%**	**95% CI**	**Numbers**^3^	**%**	**95% CI**	**Difference**	**95% CI**^4^

Specific deworming treatment	57/93	61.3	51.1, 70.6	20/47	42.6	29.5, 56.7	18.7*	-1.3, -34.7
Mebendazole (broad spectrum)	16/93	17.2	10.9, 26.1	2/47	4.3	1.2, 14.2	12.9*	1.1, 22.4
Piperazine (narrow spectrum)	28/93	30.1	21.7, 40.1	8/47	17	8.9, 30.1	13.1	-2.5, 25.9
Traditional medicine^1^	13/93	14	8.4, 22.5	10/47	21.3	12.0, 34.9	-7.3	-22.0, 5.3
Treatment not deemed necessary	36/93	38.7	29.4, 48.9	27/47	57.5	43.3, 70.5	-18.7*	-34.7, -1.3

CI = confidence interval.

*Significantly more HIV-positives remembered specific deworming treatment (including mebendazole) and fewer thought it was not necessary to treat against worms (p < 0.05).

^1^Traditional medicine breakdown (n = 23): aloe 11; herbs 2; pumpkin pips 2; dried worm 1; reeds 1; benzine 1; unspecified 5. Concerning the 11 reports of the use of aloe, it has been shown that extracts of *Aloe marlothii *have anthelmintic activity *in vitro *[50].

^2^93/170 remembered treatment detail, or non-treatment.

^3^47/65 remembered treatment detail, or non-treatment.

^4^In this column, CIs that exclude zero indicate a significant difference between the percentages for the HIV-positives and HIV-negatives (p < 0.05) [41].

## Discussion

Eighty-nine per cent of the participants were originally from the EC, which lies geographically between the WC and KwaZulu-Natal (KZN) provinces. In 2004, the antenatal HIV sero-prevalence for these three provinces was estimated to be: WC 15.4%, EC 28.0% and KZN 40.7% [19]. Infection by HIV, *Ascaris *and other helminths is widespread in all these provinces, especially under conditions of poverty in under-serviced communities [12-16]. In KZN, more species of helminths are endemic, especially in densely populated areas on the coastal plain [15].

To ensure logical development of the next phase of our research on possible immunological interaction between helminthiasis and HIV/AIDS, it is necessary to recognise that worm egg excretion status is a secondary variable that cannot be the main basis for analysis. Preliminary local data support this point with regard to *Ascaris *(which accounted for between 69% and 89% of recalls), as does a lot of published information. In the urban environment, the sero-prevalence of ascariasis can exceed egg-prevalence in children and adults. In a group of 41 women from the same community as the study groups, 51.2% were seropositive in terms of elevated *Ascaris*-specific IgE, but only 26.3% had eggs in faeces, based on several faecal samples from each adult [unpublished data]. In these adults, there was no association between seropositive status for *Ascaris *and the presence or absence of eggs in faeces. In 600 children in the same community, *Ascaris *sero-prevalence was not determined but egg-prevalence was 75% [12]. In a nearby community with better sanitation and housing, 48% of 359 children were seropositive for *Ascaris *but only 15% were egg-positive, based on two faecal samples per child [42]. Children who were egg-positive were more likely to be *Ascaris*-seropositive. In these examples from the urban CT environment, an immune response to *Ascaris *was between two- and three-fold more frequent in both adults and children than the presence of eggs in faeces. In rural areas where complications such as intestinal and duct obstructions by *Ascaris *are well-known but egg-prevalence studies have not been carried out, the position is likely to be similar.

The results make it clear that before and after sexual transmission of HIV is possible, immune responses to *Ascaris *antigens (as partly reflected by seropositivity), as well as to other endemic helminths, are likely to be detectable in a large proportion of the population studied. The presence or absence of *Ascaris *eggs in faeces is of little interpretational value because infection success or failure is determined by immune responses to the antigenic challenge, individual traits and other factors. Components of variation include the frequency of ingestion of embryonated eggs, personal hygiene, habitual behaviour (including geophagia [43]), anti-*Ascaris *immune competency or incompetency as determined genetically [34-37], and anergy [1,2,6-8]. Therefore, egg excretion is secondary to genetic, environmental and other variables, especially in adults. We contend that to evaluate potential relationships between infection by *Ascaris *(as well as other soil-transmitted helminths) and HIV/AIDS, research should focus on variables that reflect anti-*Ascaris *immune responses directly (such as cytokines [6-8,22,23,29-33] and immunoglobulins [6,8,26-28]) rather than on the presence or absence of worm eggs in excreta. When anthelmintic treatment is evaluated experimentally in terms of possible effects in relation to co-endemic disease, it is important that egg-negative people be included in deworming because they may be harbouring larval-stage or male *Ascaris *worms only (that cannot produce eggs), and/or the drug *per se *may have direct effects on the immune system [25]. In terms of recommendations by the World Health Assembly and the World Health Organisation, this procedure would be ethical and safe [15,44-47], which supports the idea of a "rapid-impact" package for simultaneously treating several neglected tropical diseases in Africa [48,49].

Recently-published research results are compatible with our rationale [27-33]. It has been shown that individuals who were persistently susceptible to infection by *Ascaris*, as demonstrated by excretion of eggs in faeces, were characterised by a weak type 2 immune response profile [30]. After age 11, a strong type 2 profile was associated with increased resistance to infection by *Ascaris *[31]. A Th2 cytokine (IL-5 or the Eosinophil Differentiation Factor) that initiates a process culminating in eosinophils attacking *Ascaris *larvae, was significantly elevated in both egg-positive and egg-negative residents where challenge from *Ascaris *antigens is inevitable due to hyperendemicity; compared to IL-5 levels in people living where *Ascaris *challenge is unusual [33]. We therefore theorise that the risk of HIV infection could be greater in egg-negative adults (than in egg-positives) if a strong type 2 response [31] down-regulates anti-viral immune factors under conditions of sustained exposure to *Ascaris *antigens and other pathogens in Africa [1,2]. In the same situation, progression to AIDS might sometimes be faster [9]; and the effectiveness of immunisation against HIV and other diseases may be impaired [1-5,23-25], especially when the sero-prevalence of ascariasis (and/or other helminthic infections) is high.

In South Africa, co-infection between *Ascaris *(and/or other helminths) and HIV/AIDS (and/or other non-helminthic diseases) is widespread [13-19,38,39,42,43]. There may be behavioural and/or sociological reasons for the apparent association between HIV-positivity and recall of intestinal helminthiasis reflected in our results, but these possibilities require further investigation. Since endemic helminthiasis will normally influence the immune environment long before heterosexual transmission of HIV (which is mainly driving the epidemic [1,17-19]) is possible, all aspects of potential disease interaction need to be researched under local circumstances in prepubertal children and in adults, but comparisons between people who have or do not have helminth eggs in their faeces must not be the primary basis for analysis. Human genetic susceptibility and resistance to ascariasis also need to be taken into consideration [34-37] because inevitable confounding of research results is often ignored.

## Conclusion

The groups that were studied were probably representative of both urban and rural populations in which the epidemic of HIV/AIDS is a huge problem. There was a long-term history of ascariasis (and probably other helminthic infections) in both the HIV-positives and the HIV-negatives. In women in the same community, and in children living where housing and sanitation are better, *Ascaris *sero-prevalence exceeded egg-prevalence by two- and three-fold, respectively. For ongoing and future analyses of possible consequences of co-infection by *Ascaris *(and/or other helminths) and HIV/AIDS (and/or other bystander conditions), comparisons must be based mainly on disease-related immunological variables. Especially in adults, comparisons cannot be based only on the presence or absence of eggs in excreta.

## Abbreviations

AIDS: acquired immunodeficiency syndrome.

CI: confidence interval.

CT: Cape Town.

EC: Eastern Cape province of South Africa.

HIV: human immunodeficiency virus type 1.

HIV/AIDS: infection by HIV that has progressed to AIDS.

HTLV-1: human T cell lymphotropic virus type 1.

KZN: KwaZulu-Natal province of South Africa.

NSD: no significant difference (used in tables).

WC: Western Cape province of South Africa.

## Competing interests

The authors declare that they have no competing interests.

## Authors' contributions

VJ Adams: overall planning, drafting questionnaire, community liaison, counselling, medical liaison, field management, data processing, checking text.

MB Markus: main editor, writing, literature searching, helminthology consultant, English language consultant.

ZL Kwitshana: community liaison, drafting and testing questionnaire, checking text.

MA Dhansay: medical consultant, community liaison, ethics consultant, checking text.

L van der Merwe: statistician, checking text.

G Walzl: immunology consultant.

JE Fincham: main writer, co-editor, overall planning, literature searching, community liaison, research liaison, drafting questionnaire, analysis of results.

## Pre-publication history

The pre-publication history for this paper can be accessed here:


